# Supplementary material for: Organogenesis in a Broad Spectrum of Grape Genotypes and Agrobacterium-Mediated Transformation of the Podarok Magaracha Grapevine Cultivar
Source: Plants (Basel). 2024 Oct 3;13(19):2779. doi: 10.3390/plants13192779 (PMC11478747; doi:10.3390/plants13192779)
Supplement: Supplementary file 1 [file plants-13-02779-s001.zip › plants-3059555-supplementary.pdf]

Supplementary Table S1 Variations in media for regeneration of 22 studies grapevine genotypes at presence TDZ+IBA (regeneration efficiency data is presented in %).

	Genotype	petiole fragments									internode fragments									leaf fragments									Average
		0.5 mg l-1 TDZ			1.0 mg l-1 TDZ			1.5 mg l-1 TDZ			0.5 mg l-1 TDZ			1.0 mg l-1 TDZ			1.5 mg l-1 TDZ			0.5 mg l-1 TDZ			1.0 mg l-1 TDZ			1.5 mg l-1 TDZ			
		0.01 mg l-1 IBA	0.05 mg l-1 IBA	0.10 mg l-1 IBA	0.01 mg l-1 IBA	0.05 mg l-1 IBA	0.10 mg l-1 IBA	0.01 mg l-1 IBA	0.05 mg l-1 IBA	0.10 mg l-1 IBA	0.01 mg l-1 IBA	0.05 mg l-1 IBA	0.10 mg l-1 IBA	0.01 mg l-1 IBA	0.05 mg l-1 IBA	0.10 mg l-1 IBA	0.01 mg l-1 IBA	0.05 mg l-1 IBA	0.10 mg l-1 IBA	0.01 mg l-1 IBA	0.05 mg l-1 IBA	0.10 mg l-1 IBA	0.01 mg l-1 IBA	0.05 mg l-1 IBA	0.10 mg l-1 IBA	0.01 mg l-1 IBA	0.05 mg l-1 IBA	0.10 mg l-1 IBA	
	Vitis vinifera conv. *occidentalis*																												
1	Cabernet Sauvignon	0a	0a	0a	0a	0a	0a	0a	0a	0a	0a	1,7a	0a	0a	0a	0a	0a	0a	0a	0a	0a	0a	0a	0a	0a	0a	0a	1,7a	0.13
2	Chardonnay	0a	0a	0a	0a	0a	0a	0a	0a	0a	0a	0a	0a	0a	0a	0a	0a	0a	0a	0a	0a	0a	0a	0a	0a	0a	0a	0a	0
3	Merlot	0a	0a	0a	0a	0a	0a	0a	0a	1,7 a	0a	0a	0a	0a	0a	0a	0a	0a	0a	0a	0a	1,7 a	1,7 a	0a	0a	0a	0a	0a	0.19
4	Pinot Noir	0a	0a	0a	0a	0a	0a	0a	0a	0a	0a	0a	0a	0a	0a	0a	0a	0a	0a	0a	0a	1,7 a	1,7 a	0a	0a	0a	0a	0a	0.13
5	Pinot Gris	0a	0a	0a	0a	0a	0a	0a	0a	0a	0a	0a	0a	0a	0a	0a	0a	0a	0a	0a	0a	0a	0a	0a	0a	0a	0a	0a	0
6	Aligote	0a	1,2 a	1,2 a	0a	1,2 a	1,2 a	0a	0a	0a	0a	1,2 a	0a	0a	0a	10,0 b	0a	4,6 ab	0a	0a	0a	2,4 ab	0a	0a	1,2 a	0a	0a	0a	0.9
7	Syrah	0a	0a	5.3abcde	0a	0a	9.8cde	0a	9,8bcde	0a	0a	0a	0a	0a	0a	9.8de	0a	5.3 abcde	0a	0a	0a	0a	0a	0a	1.7 abcde	0a	1.7 abcde	9.8 e	2.0
8	Bastardo	0a	1,7a	1,7a	0a	0a	1,7a	3,5a	3,5a	0a	0a	0a	1,7a	0a	0a	0a	0a	1,7a	1,7a	0a	0a	0a	0a	0a	0a	0a	0a	0a	0.64
	Vitis vinifera conv. *orientalis*																												
9	Muscat Blanc	0a	0a	0a	0a	0a	0a	0a	0a	0a	0a	0a	0a	0a	0a	0a	0a	0a	0a	0a	0a	0a	0a	0a	0a	0a	0a	0a	0
	Vitis vinifera x American *Vitis* hybrids																												
10	Citroniy Magaracha	0a	0a	0a	0a	0a	0a	0a	0a	0a	0a	0a	0a	0a	0a	0a	0a	0a	0a	0a	0a	0a	0a	0a	0a	0a	0a	0a	0
11	Veles	0a	1,7 a	0a	0a	0a	0a	0a	0a	0a	0a	0a	0a	0a	0a	0a	0a	0a	0a	1,7 a	1,7 a	1,7 a	1,7 a	0a	0a	0a	1,7 a	0a	0.38
12	Muscat Crima	10 c	0a	0a	0a	1,2 ab	0a	0a	0a	0a	1,2 ab	0a	0a	0a	1,2 ab	3,8 bc	0a	0a	0a	0a	0a	0a	0a	0a	0a	0a	0a	0a	0.64
13	Magarach No. 112	0a	1,7 a	0a	0a	0a	0a	0a	0a	0a	0a	0a	0a	0a	0a	0a	0a	0a	0a	1,7 a	1,7 a	1,7 a	1,7 a	0a	0a	0a	1,7 a	0a	0.38
14	Yaltinskiy bessemyannyy	0a	0a	15.8 d	0a	2.4 abcd	1.2 abcd	0a	2.4 abcd	1.2 abcd	4.6 abcd	9.2 abcd	1.2 abcd	0a	13.1bcd	2.4 abcd	0a	0a	4.6 abcd	0a	0a	13.9 cd	0a	0a	3.7 abcd	0a	1.2 abcd	0a	2.85
15	Krymskiy biser	1.7a	1.7a	1.7a	0a	0a	1.7a	0a	0a	0a	0a	0a	1.7a	0a	0a	3.5a	0a	0a	0a	0a	0a	3.5a	0a	0a	0a	0a	0a	0a	0.57
16	Academic Avidzba	0a	0a	0a	0a	0a	1,7 a	0a	1,7 a	0a	0a	0a	0a	0a	0a	0a	0a	0a	0a	0a	0a	0a	0a	0a	9,8 b	0a	1,7 a	1,7 a	0,79
17	Liviya	0a	0a	0a	1,7a	0a	0a	0a	3,5a	0a	0a	0a	0a	0a	0a	0a	0a	1,7a	0a	0a	0a	0a	0a	0a	0a	0a	1,7 a	0a	0.32
18	Kefesia Magaracha	0a	0a	0a	0a	0a	1.7 a	0a	0a	0a	0a	0a	0a	0a	1.7 a	0a	0a	0a	0a	0a	0a	0a	0a	0a	9.8 bc	0a	0a	9.8 c	0.85
19	Podarok Magaracha	23,2 cdef	29.9 def	26.8 cdef	33.4 ef	33.4 f	29.9 def	26.8 cdef	33.4 f	29.9 def	29.9 def	33.4 f	29.9 def	16.5 c	33.4 f	29.9 def	20.1 cd	33.4 f	29.9 def	1.7 a	50.0 g	98.3 ijk	6.7 b	83.5 h	**100 k**	20.0 cd	100 jk	100 k	40.12
20	Sphinx	0a	0a	0a	0a	0a	0a	0a	0a	0a	0a	0a	0a	0a	0a	0a	0a	0a	0a	0a	0a	0a	0a	0a	0a	0a	0a	0a	0
21	Ruta	26.6 defgh	46.6 ghijkl	62.0 jklm	28.4 efghi	**87.0 m**	50.0 hijkl	29.6 efghij	60.2ijkl	66.8lm	19.4 bcdefg	43.2 ghijkl	53.3 hijkl	49.9 hijkl	64.1klm	43.4 ghijkl	39.4 ghijkl	50.0 hijkl	50.0 hijkl	1.2a	33.3 fghijk	29.7 efghi	1.2a	26.7 cdefgh	2.4ab	10.0 abcdef	6.7 abcde	4.6 ab	36.51
	Non-*vinifera Vitis* species and hybrids																												
22	*V. berlandieri* x *V. riparia* 'Kober 5BB	1,2 ab	0a	6.7 abcd	22.5 cd	4.6 abcd	9.2 abcd	25.5 d	2.4 abc	6.7 abcd	1.2 ab	2.4 abc	1.2 ab	8.7 abcd	4.6 abcd	0a	19.4 bcd	2.4 abc	4.6 abcd	19.4 bcd	2.4 abc	4.6 abcd	1.2 ab	0a	0a	0a	0a	0a	5.59

Each genotype was analyzed by three-way (1 - explant type, 2 - TDZ concentration, 3 - IBA concentration) analysis of variance separately from other genotypes. Different letters in a column indicate significant differences in variant data.

Supplementary Table S2 Variations in media for regeneration of 22 studies grapevine genotypes at presence BA+IBA (regeneration efficiency data is presented in %).

№	Genotype	petiole fragments									internode fragments									leaf fragments									Average
		2.0 mg l^{-1} BA			2.5 mg l^{-1} BA			3.0 mg l^{-1} BA			2.0 mg l^{-1} BA			2.5 mg l^{-1} BA			3.0 mg l^{-1} BA			2.0 mg l^{-1} BA			2.5 mg l^{-1} BA			3.0 mg l^{-1} BA			
		0.05 mg l^{-1} IBA	0.10 mg l^{-1} IBA	0.15 mg l^{-1} IBA	0.05 mg l^{-1} IBA	0.10 mg l^{-1} IBA	0.15 mg l^{-1} IBA	0.05 mg l^{-1} IBA	0.10 mg l^{-1} IBA	0.15 mg l^{-1} IBA	0.05 mg l^{-1} IBA	0.10 mg l^{-1} IBA	0.15 mg l^{-1} IBA	0.05 mg l^{-1} IBA	0.10 mg l^{-1} IBA	0.15 mg l^{-1} IBA	0.05 mg l^{-1} IBA	0.10 mg l^{-1} IBA	0.15 mg l^{-1} IBA	0.05 mg l^{-1} IBA	0.10 mg l^{-1} IBA	0.15 mg l^{-1} IBA	0.05 mg l^{-1} IBA	0.10 mg l^{-1} IBA	0.15 mg l^{-1} IBA	0.05 mg l^{-1} IBA	0.10 mg l^{-1} IBA	0.15 mg l^{-1} IBA	
Vitis vinifera conv. *occidentalis*																													
1	Cabernet Sauvignon	0a	0a	0a	0a	0a	0a	0a	0a	0a	0a	0a	0a	0a	0a	0a	0a	0a	0a	0a	0a	0a	0a	0a	0a	0a	0a	0a	0
2	Chardonnay	0a	0a	0a	0a	0a	0a	0a	0a	0a	0a	0a	0a	0a	0a	0a	0a	0a	0a	0a	0a	0a	1,1a	0a	0a	1,1a	0a	0a	0.08
3	Merlot	0a	0a	0a	0a	0a	0a	0a	0a	0a	0a	0a	0a	0a	0a	0a	0a	0a	0a	0a	0a	0a	0a	0a	0a	0a	0a	0a	0
4	Pinot Noir	0a	0a	0a	0a	0a	0a	0a	0a	0a	0a	0a	0a	0a	0a	0a	0a	0a	0a	0a	0a	0a	0a	0a	0a	0a	0a	0a	0
5	Pinot Gris	0a	0a	0a	0a	0a	0a	0a	0a	0a	0a	0a	0a	0a	0a	0a	0a	0a	0a	0a	0a	0a	0a	0a	0a	0a	0a	0a	0
6	Aligote	0a	0a	1,1a	0a	0a	0a	0a	0a	0a	0a	0a	0a	0a	0a	0a	0a	0a	0a	0a	0a	0a	0a	0a	0a	0a	0a	0a	0.04
7	Syrah	1.7abcde	0a	1.7abcde	1.7abcde	0a	1.7abcde	6.6abcde	1.7abcde	3.5abcde	0a	0a	1.7abcde	1.7abcde	0a	0a	1.7abcde	0a	1.7abcde	1.6abcde	5.2abcde	12.4de	11.5cde	5.6abcde	7.8abcde	9.9bcde	2.7abcde	15.3e	3.61
8	Bastardo	23,5gh	23,2 defgh	0a	**33,4h**	23,2 efgh	19.6 cdefgh	5,3 abcdefg	23,2 gh	15.3 bcdefgh	6,7 abcdefg	0a	0a	0a	0a	3.5 abc	0a	0a	1.7 ab	0a	0a	0a	0a	0a	0a	0a	0a	0a	6.61
Vitis vinifera conv. *orientalis*																													
9	Muscat Blanc	0a	0a	0a	0a	0a	0a	0a	0a	0a	0a	0a	0a	0a	0a	0a	0a	0a	0a	0a	0a	0a	0a	0a	0a	0a	0a	0a	0
Vitis vinifera x American *Vitis* hybrids																													
10	Citroniy Magaracha	0a	0a	0a	0a	0a	0a	0a	0a	0a	0a	0a	0a	0a	0a	0a	0a	0a	0a	0a	0a	0a	0a	0a	0a	1,1a	0a	0a	0.04
11	Veles	0a	0a	0a	0a	0a	0a	0a	0a	0a	0a	0a	0a	0a	0a	0a	0a	0a	0a	0a	0a	0a	0a	0a	0a	0a	0a	0a	0
12	Muscat Crima	0a	0a	0a	0a	0a	0a	0a	0a	0a	0a	0a	0a	0a	0a	0a	0a	0a	0a	0a	0a	1.7b	0a	3.5bc	0a	0a	0a	12.6c	0.66
13	Magarach No. TT2	0a	0a	0a	0a	0a	0a	0a	0a	0a	0a	0a	0a	0a	0a	0a	0a	0a	0a	0a	0a	0a	0a	0a	0a	1,1a	0a	0a	0.04
14	Yaltinskiy bessemyannyy	0a	1.7a	1.7a	0a	0a	3.5a	1.7a	1.7a	0a	0a	1.7a	0a	1.7a	0a	1.7a	0a	1.7a	0a	0a	9.6a	19.5ab	6.6a	3.5a	9.6a	12.5a	**50c**	7.2a	5.0
15	Krymskiy biser	0a	0a	0a	0a	0a	0a	0a	0a	0a	0a	0a	0a	0a	0a	0a	0a	0a	0a	20.0 cdefgh	29.9 hi	39.2 ij	23.3 efgh	23.3 defgh	**53.4 j**	26.7 ghi	26.7 fghi	3.5 b	9.11
16	Academic Avidzba	0a	0a	0a	0a	0a	0a	0a	0a	0a	0a	0a	0a	0a	0a	0a	0a	0a	0a	1.7ab	26.4 defg	36.5gh	3.5b	33.4 efgh	36.5fgh	0a	20.0cd	43.2h	7.54
17	Liviya	0a	0a	0a	0a	0a	0a	0a	0a	0a	0a	0a	0a	0a	0a	0a	0a	0a	0a	0a	0a	0a	0a	0a	0a	0a	0a	0a	0
18	Kefesia Magaracha	0a	0a	0a	0a	0a	0a	0a	0a	0a	0a	0a	0a	0a	0a	0a	0a	0a	0a	0a	0a	1,1a	0a	0a	13.0b	0a	3,7a	1,1a	0.7
19	Podarok Magaracha	19,5f	13.3def	0a	16.5ef	1.5abcd	1.7abcd	13.3cdef	1.7abcd	0a	9.7bcdef	0a	0a	3.5abcde	1.7abcd	1.7abcd	6.6 bcdef	0a	0a	**87.4o**	63.3 ijklmn	63.6klmn	76.8no	53.3 ghijklmn	63.3 jklmn	66.8mn	53.3 hijklmn	63.6lmn	25.26
20	Sphinx	0a	0a	0a	0a	0a	0a	0a	0a	0a	0a	0a	0a	0a	0a	0a	0a	0a	0a	0a	0a	0a	0a	0a	0a	0a	0a	0a	0

21	Ruta	1,1a	0a	3.5a	13.0a	3.5a	1.1a	0a	1.1a	3.5a	1.1a	0a	0a	3.5a	1.1a	0a	0a	0a	0a	1.1a	0a	0a	0a	10a	0a	0a	0a	0a	1.61
	Non-*vinifera Vitis* species and hybrids																												
22	*V. berlandieri* x *V. riparia* 'Kober 5BB	29.5 efghi	29.5 fghi	**53.3i**	46.5hi	43.2 ghi	25.4 defghi	19.5 cdefghi	19.5 cdefghi	17.9 bcdefghi	15.2 bcdefgh	12.5 bcdefgh	26.4fghi	25.4efghi	23.2 cdefghi	12.5 bcdefgh	3.5 abcdef	9.7 bcdefg	5.3 abcdef	4.5abcdef	4.5 abcdef	9.2 bcdef	0a	9.2 bcdef	1.1abc	1.1abc	0a	1.1abc	16.62

Each genotype was analyzed by three-way (1 - explant type, 2 - BA concentration, 3 - IBA concentration) analysis of variance separately from other genotypes. Different letters in a column indicate significant differences in variant data

Supplementary Table S3 Parameters of the TDZ+IBA logistic regression model

№	Genotype	Parameters	Coefficients	Std. err.	p-value	Confidence Intervals	Psevdo R-squ. (McFadden's)
1	Aligote	explant type	-1,075813	0,301667	0,000362	[-1.667069, -1.667069]	-0,085987
		cytokinin concentration	-2,785584	0,670886	0,000033		
		auxin concentration	4,663130	6,049785	**0,440830**		
2	Syrah	explant type	-2,528267	0,767635	0,000989	[-4.032803, -4.032803]	-0,126643
		cytokinin concentration	-1,707898	0,945401	**0,070835**		
		auxin concentration	6,474639	9,574129	**0,498873**		
3	Muscat Crima	explant type	-1,196788	0,430442	0,005430	[-2.040439, -2.040439]	0,067525
		cytokinin concentration	-2,393576	0,860884	0,005430		
		auxin concentration	-12,178775	9,605900	**0,204854**		
4	Yaltinskiy bessemyannyy	explant type	-0,683780	0,169451	0,000055	[-1.015897, -1.015897]	-0,006524
		cytokinin concentration	-2,493431	0,404597	7,15E-10		
		auxin concentration	7,962301	3,968661	0,044825		
5	Krymskiy biser	explant type	19,943478	21040,252376	**0,999244**	[-41218.193404, -41218.193404]	0,434091
		cytokinin concentration	-196,988631	128991,903847	**0,998782**		
		auxin concentration	353,316768	132475,614718	**0,997872**		
6	Academic Avidzba	explant type	-0,751038	0,948493	**0,428464**	[-2.610051, -2.610051]	-0,490608
		cytokinin concentration	-7,117201	3,501434	0,042088		
		auxin concentration	-1,389340	19,192869	**0,942293**		
7	Liviya	explant type	-2,490263	0,740106	0,000766	[-3.940844, -3.940844]	-0,119943
		cytokinin concentration	-0,279428	0,783247	**0,721275**		
		auxin concentration	-22,548394	12,854915	**0,079419**		
8	Kefesia Magaracha	explant type	-1,000489	0,319280	0,001727	[-1.626267, -1.626267]	-0,407034
		cytokinin concentration	-2,742799	0,721845	0,000145		
		auxin concentration	-1,518739	6,749145	**0,821958**		
9	Podarok Magaracha	explant type	0,118107	0,070803	0,095296	[-0.020665, -0.020665]	0,004653
		cytokinin concentration	-0,923980	0,147801	4,07E-10		
		auxin concentration	6,797875	1,892239	0,000328		
10	Ruta	explant type	-0,721608	0,079970	0,000000	[-0.878346, -0.878346]	0,083986
		cytokinin concentration	0,278206	0,144368	**0,053973**		
		auxin concentration	9,842349	1,964079	0,000001		
11	V. berlandieri x V. riparia'Kober 5BB	explant type	-0,853461	0,143942	3,04E-09	[-1.135582, -1.135582]	0,034165
		cytokinin concentration	-0,238719	0,243143	0,326195		
		auxin concentration	-15,102131	3,756225	0,000058		

Supplementary Table S4 Parameters of the BA+IBA logistic regression model

№	Genotype	Parameters	Coefficients	Std. err.	p-value	Confidence Intervals	Psevdo R-squ. (McFadden's)
1	Syrah	explant type	0,592018	0,205471	0,003961	[0.189302, 0.189302]	-0,027590
		cytokinin concentration	-1,737857	0,261333	2,93E-11		
		auxin concentration	-4,952274	3,821163	**0,194971**		
2	Bastardo	explant type	-2,993461	0,538608	2,73E-08	[-4.049113, -4.049113]	0,269831
		cytokinin concentration	0,680195	0,249114	0,006325		
		auxin concentration	-4,816486	3,978541	**0,226043**		
3	Muscat Crima	explant type	0,410159	0,518266	0,428707	[-0.605624, -0.605624]	-0,177233
		cytokinin concentration	-2,616204	0,701001	0,000190		
		auxin concentration	1,787454	10,117629	**0,859770**		
4	Yaltinskiy bessemyannyy	explant type	0,682062	0,214395	0,001466	[0.261856, 0.261856]	-0,067514
		cytokinin concentration	-1,657203	0,262050	2,55E-10		
		auxin concentration	-6,301054	4,156811	**0,129560**		
5	Krymskiy biser	explant type	1,757930	0,221935	2,36E-15	[1.322946, 1.322946]	0,218466
		cytokinin concentration	-2,698767	0,279263	4,29E-22		
		auxin concentration	-2,312469	3,282268	**0,481100**		
6	Academic Avidzba	explant type	1,266828	0,200711	2,76E-10	[0.873442, 0.873442]	0,133190
		cytokinin concentration	-2,719931	0,277771	1,22E-22		
		auxin concentration	9,089844	3,433949	0,008120		
7	Kefesia Magaracha	explant type	0,692732	0,385134	**0,072069**	[-0.062116, -0.062116]	-0,061796
		cytokinin concentration	-2,728375	0,536350	3,64E-07		
		auxin concentration	4,687825	7,195646	**0,514736**		
8	Podarok Magaracha	explant type	2,144333	0,173855	5,94E-35	[1.803584, 1.803584]	0,327397
		cytokinin concentration	-1,927014	0,174928	3,20E-28		
		auxin concentration	-14,058088	2,524254	2,56E-08		
9	Ruta	explant type	-0,935032	0,699465	**0,181294**	[-2.305958, -2.305958]	0,106401
		cytokinin concentration	-0,440038	0,613693	**0,473353**		
		auxin concentration	-33,262074	19,203278	**0,083255**		
10	V. berlandieri x V. riparia 'Kober 5BB	explant type	-0,848027	0,131852	1,26E-10	[-1.106452, -1.106452]	0,095690
		cytokinin concentration	-0,241019	0,126289	0,056329		
		auxin concentration	3,807618	2,383685	**0,110184**		
